# Determination of the Key Resistance Gene Analogs Involved in *Ascochyta rabiei* Recognition in Chickpea

**DOI:** 10.3389/fpls.2019.00644

**Published:** 2019-05-17

**Authors:** Ziwei Zhou, Ido Bar, Prabhakaran Thanjavur Sambasivam, Rebecca Ford

**Affiliations:** Environmental Futures Research Institute, School of Environment and Science, Griffith University, Nathan, QLD, Australia

**Keywords:** Resistance Gene Analogs, ascochyta blight, chickpea, host resistance, expression profiling

## Abstract

Chickpea (*Cicer arietinum* L.) is an important cool season food legume, however, its production is severely constrained by the foliar disease Ascochyta blight caused by the fungus *Ascochyta rabiei* (syn. *Phoma rabiei*). Several disease management options have been developed to control the pathogen, including breeding for host plant resistance. However, the pathogen population is evolving to produce more aggressive isolates. For host resistance to be effective, the plant must quickly recognize the pathogen and instigate initial defense mechanisms, optimally at the point of contact. Given that the most resistant host genotypes display rapid pathogen recognition and response, the approach taken was to assess the type, speed and pattern of recognition via Resistance Gene Analog (RGA) transcription among resistant and susceptible cultivated chickpea varieties. RGAs are key factors in the recognition of plant pathogens and the signaling of inducible defenses. In this study, a suite of RGA loci were chosen for further investigation from both published literature and from newly mined homologous sequences within the National Center for Biotechnology Information (NCBI) database. Following their validation in the chickpea genome, 10 target RGAs were selected for differential expression analysis in response to *A. rabiei* infection. This was performed in a set of four chickpea varieties including two resistant cultivars (ICC3996 and PBA Seamer), one moderately resistant cultivar (PBA HatTrick) and one susceptible cultivar (Kyabra). Gene expression at each RGA locus was assessed via qPCR at 2, 6, and 24 h after *A. rabiei* inoculation with a previously characterized highly aggressive isolate. As a result, all loci were differentially transcribed in response to pathogen infection in at least one genotype and at least one time point after inoculation. Among these, the differential expression of four RGAs was significant and consistently increased in the most resistant genotype ICC3996 immediately following inoculation, when spore germination began and ahead of penetration into the plant’s epidermal tissues. Further *in silico* analyses indicated that the differentially transcribed RGAs function through ADP-binding within the pathogen recognition pathway. These represent clear targets for future functional validation and potential for selective resistance breeding for introgression into elite cultivars.

## Introduction

Chickpea (*Cicer arietinum* L.) is a staple cool season food legume, important in the Indian sub-continent, West Asia, North Africa and grown as a high-return cash export crop in Australia and North America (Du et al., 2012). However, production is seriously constrained by fungal disease Ascochyta blight, which is the most frequent and devastating disease of chickpea crops worldwide (Sagi et al., 2017). The fungus *Ascochyta rabiei* (syn. *Phoma rabiei*), can infect all parts of the plant above ground, and at any growth stage (Sharma and Ghosh, 2016).

Australia is the second largest global producer and exporter of chickpea (ABARE report from February 2, 2016), while India is the largest chickpea producer, whose production dwarfs that of all other countries. The first recorded *A. rabiei* epidemic in Australia occurred in 1998 (Du et al., 2012). With growing market demand and cash return, production in northern New South Wales and southern and central Queensland has recently increased. This has led to significantly increased risk from *A. rabiei* due to complacency in disease management best practice from novice growers and the potential for wetter winters than in southern growing regions. During the 2012–2014 seasons, the high rainfall in these northern regions led to widespread *A. rabiei* epidemics; and highly aggressive clonal isolates destroyed crops of the most resistant cultivars despite repeated fungicide applications (Moore et al., 2015b). Despite the presence of the teleomorph elsewhere, the Australian population is asexual, reliant on mutational events for favorable selection and potential adaptation (Leo et al., 2015). The emergence of growing numbers of highly aggressive isolates across the growing regions indicated sufficient genetic diversity within the clonal population to select for ability to overcome the fungicides and host resistance genes employed (Mehmood et al., 2017).

Since there appears to be a growing potential for *A. rabiei* to evolve new pathotypes with high aggressiveness (Mehmood et al., 2017), it is important for breeders to be able to select for germplasm with the best and most stable resistance. This may in part be informed by understanding the functional pathogen recognition mechanisms, of which RGAs play a key role and are responsible for the onward signaling and activating of plant defense responses shown to be involved in many plant pathosystems (Grant et al., 1998).

Resistance Gene Analogs (RGAs) are a large gene family with conserved domains and structural features that enable classification into either nucleotide binding site leucine rich repeat (NBS-LRR) or transmembrane leucine rich repeat (TM-LRR) sub-families. They function mainly as intracellular receptors that perceive the presence of pathogen effectors by direct binding of the pathogen effector proteins, or by monitoring the modification of host proteins after associating with the pathogen, to activate multiple defense signal transductions to restrict pathogen growth (Sagi et al., 2017). Emerging evidence indicates that an intermediate vesicle-type exosomal body is involved in delivering the molecules that initiate the chickpea signaling for defense to necrotrophic fungi (Boevink, 2017). In the Chickpea – *A. rabiei* pathosystem, RGAs are predicted to recognize the fungus and then induce signaling of defense molecules previously identified by Coram and Pang (2006), leading to resistance in several commonly grown chickpea cultivars (i.e., PBA Seamer).

Subsequent plant defense responses are complex and diverse at the genomic level, the expression of transcription factors and protein kinases, as well as the increase in cytosolic calcium are all involved in defense signaling (Grant and Mansfield, 1999). Moreover, the speed and coordination of the host’s perception of the pathogen, signal transduction and transcriptional activation are also vital to successful defense. In the study by Coram and Pang (2006), 13.6% of chickpea complementary DNAs (cDNAs) evaluated by microarray were differentially expressed in response to *A. rabiei*. Further, the kinetics of differential expression after inoculation of *A. rabiei* highlighted the differential timing of pathogen recognition and subsequent transcriptional changes associated with the *A. rabiei* defense response (Coram and Pang, 2005a,b; Leo et al., 2016).

Although the earlier studies identified some key defense-related mechanisms, the underlying pathogen recognition factors were not elucidated. In addition, the defense of chickpea to ascochyta blight is multigenic and governed by resistance-quantitative trait loci (R-QTL) with many QTLs for *A. rabiei* resistance identified on multiple linkage groups (Santra et al., 2000; Leo et al., 2016; Sagi et al., 2017). According to Sagi et al. (2017), 121 NBS-LRR genes are associated to R-QTL for *A. rabiei*. Subsequent assessment of their expression levels at 12, 24, 48, and 72 hpi revealed several RGAs that are deemed functional in early pathogen recognition. However, together with those previously identified by Leo et al. (2015), they represent only a subset of the possible recognition factors and their activities at earlier and crucial time points are still unknown. Characterization and functional assessment of a wider range of RGAs at the “pre-penetration” and “during penetration” stages will provide essential information for future targeted breeding of varieties able to quickly recognize and respond to this devastating pathogen. Therefore, the aims of this study were to: (1) Identify RGA candidates present in the chickpea genome through published literature searches and sequence analyses; (2) Validate the presence of RGA candidates within key resistant chickpea genotypes; (3) Assess the putative function of the RGA candidates via transcription in response to an aggressive isolate of *A. rabiei* at biologically important early interaction stages; and (4) Further characterize the putative function of the most responsive RGA candidates through predictive *in silico* analyses.

## Materials and Methods

### Target RGA Loci and Development of PCR Markers

Five sequences, representative of three RGA classes which were previously characterized and considered putatively functional in resistance to fusarium wilt, rust, and ascochyta blight (Palomino et al., 2009), were initially chosen for further assessment. These included RGAs of class 01, previously detected in faba bean and RGAs of classes 02 and 03, previously detected in chickpea (Palomino et al., 2009). Additionally, four chickpea NBS-LRR RGA loci were chosen from Leo et al. (2016). Finally, three RGA sequences, reported to be up-regulated in response to *A. rabiei*, were chosen from Sagi et al. (2017). Simultaneously, thirteen RGA sequences were sought from chickpea sequences deposited to the NCBI database^1^. The 13 sequences were chosen because they represented the breadth of the RGA families and they were unanimously identifiable from the existing database. Seeking and assigning of putative RGAs was performed using known motifs for specific RGA classes (NBS-LRR family) with a 99% of within-class identity threshold, while the motif information was referenced from Sekhwal et al. (2015).

PCR primers flanking the selected RGA loci were designed using Primer3web (version 4.0.0^2^) with the following criteria: melting temperature (Tm) of 59 ± 3°C, and PCR amplicon size of 150–300 base pair (bp), primer length of 18–23 nucleotides and GC content of 40–60%. Primers were synthesized by SIGMA-ALDRICH.

### Plant Material and Fungal Isolates

Four chickpea genotypes with differentially known disease reactions to *A. rabiei* were used; ICC3996, PBA Seamer, PBA HatTrick, and Kyabra (Table 1). It is worth mentioning that even the resistance varieties are evaluating show substantial disease symptoms under many typical field epidemic situations. Seed was obtained from the National Chickpea Breeding Program, Tamworth, NSW, Australia. Seedlings were grown in 15 cm diameter pots containing commercial grade potting mix (Richgro premium mix), with 5 seed per pot/replication (six replicates per host genotype and isolate). Plants were grown in a controlled growing environment (CGE) maintained at 22 ± 1°C with a 16/8 h (light/dark) photoperiod for 14 days until inoculation. The *A. rabiei* isolate FT13092-1 used in this experiment was collected in 2013 from Kingsford, South Australia (by Dr. Jenny Davidson of the South Australian Research and Development Institute). Isolate FT13092-1 is highly aggressive on PBA HatTrick, Kyabra, and is moderately aggressive on ICC3996 (Grains Research and Development Corporation annual report for project #UM00052; R. Ford pers. comm.). The single-spored isolate was cultured on V8 juice agar and maintained in the incubator for 14 days at 22 ± 2°C with a 12/12 h near-UV light irradiation (350–400 nm)/dark photoperiod.

**Table 1:** Chickpea genotypes and disease ratings to *A. rabiei* in Australia.

Genotype/Cultivar	Disease rating	Pedigree	References
ICC 3996	Resistant (R)	ICC 3996 is a landrace	Moore et al., 2015b
PBA Seamer	Resistant (R)	PBA Seamer (evaluated as CICA0912) was developed by the PBA chickpea breeding program from a cross between the breeding line 98081-3024 and PBA HatTrick	Pulse Breeding Australia, 2018
PBA HatTrick	Moderately resistant/Resistant (MR/R)	JIMBOUR/ICC14903	Plett et al., 2016; Pulse Breeding Australia, 2018
Kyabra	Susceptible (S)	Amethyst//946-31/Barwon//Lasseter/940-26//946-31/Norwin//8507-28H//Amethyst//T1069/8507-28H//946-31	Plett et al., 2016; Pulse Breeding Australia, 2018

### Preparation of Inoculum and Bioassay

Inoculum was prepared by adding 10 mL of sterile distilled water to the cultured plates and scraping the pycnidia with a sterile bent glass rod to release pycnidiospores. The spore suspension was then filtered through muslin cloth and the final spore concentration was adjusted to 10^5^ spores⋅mL^−1^. Since three replications are sufficient to show significant consistency, three replicates (three pots) of 14-day-old seedlings were sprayed using an air-pressured hand-held sprayer with a fine mist of prepared inoculum until run-off and labeled as treated groups. Another three replicates were sprayed with sterile water and labeled as untreated groups. Tween 20 (0.02% v/v) was added to the inoculum and water as a surfactant. All plants were covered with inverted plastic cups immediately after the inoculation according to the mini-dome technique (Chen et al., 2005) to ensure maximum humidity and darkness to induce optimum spore germination (Sambasivam et al., 2017) maintained in a CGE at 22 ± 1°C. The main stems and young leaf tissues from treated and untreated groups were collected at 2, 6, and 24 hpi into 25 mL falcon tubes, snap frozen in liquid N_2_, and stored at −80°C until processing. Following collection of foliar tissue for transcript analyses at each of the time points from individual plants, the remaining plant was left under the bioassay conditions to develop disease symptomology to confirm a viable infection had occurred.

### RNA Extraction, cDNA Preparation, and Differential Expression via RT-qPCR

RNA was extracted from the leaf and stem tissues of inoculated and uninoculated samples using a NucleoSpin^®^ RNA Plant kit (Macherey-Nagel, Germany) according to the manufacturer’s instructions. The RNA sample purity was assessed by reading the OD_260_/OD_280_ absorption ratio using a Nano drop spectrometer (ND-1000). Total RNA (1 μg) of each sample was used for Genomic DNA (gDNA) elimination and reverse transcription using a PrimeScript^TM^ RT reagent Kit with gDNA Eraser (Perfect Real Time; Takara Bio, United States). The quality of cDNA and absence of gDNA were evaluated through PCR by using the primer pair used to amplify the chickpea reference gene (CAC) from Reddy et al. (2016) which produced an amplicon that spanned intron-exon boundaries. The expected amplification product size was 110 bp and this was validated by electrophoresis. The cDNA samples were then diluted (1:50) with DNase/RNase free water for RT-qPCR. Each primer pair was assessed for PCR amplification on gDNA and cDNA samples. In addition, three reference genes (ABCT, UCP, and CAC) were selected from Reddy et al. (2016) and used as Inter-Run Calibrators (IRC), since they were previously shown to be stably expressed across many chickpea varieties. All primer sequences designed are listed in Supplementary Figure 1 and Supplementary Table 1. The PCR efficiency of each primer pair was evaluated by using serially diluted cDNA samples (10^0^, 10^−1^, 10^−10^, 10^−100^, 10^−1000^). Bio-Rad CFX Manager 3.1 software (Bio-Rad, CA, United States) and a custom R script were used to calculate the correlation coefficient (*R*^2^), slope value, and PCR amplification efficiency (E) of each primer pair combination.

A SYBR^®^ Premix Ex Taq^TM^ II (TIi RNaseH Plus) kit was used for assessing target gene expression using optical 96 well plates on a BIO-RAD CFX96 real-time PCR detection system (Bio-Rad laboratories) and reactions were prepared according to the manufacturer’s instructions. The PCR reactions were performed in a total volume of 25 μL containing 12.5 μL of 2x SYBR^®^ Premix Ex Taq^TM^ II (TIi RNaseH Plus), 0.4 μM of each primer, and 2 μL of diluted cDNA template. The reaction conditions were set as 30 s at 95°C (initial denaturation); followed by 40 cycles of 95°C for 5 s, 60°C for 30 s (fluorescence reading), and then followed by a melt curve analysis at 65–95°C every 0.5°C for 10 s. All reactions were carried out in technical duplicates. If variations between duplicates were significant, a triplicate was performed, and the two closest data points were taken. IRC were used in every single plate, because all samples in this experiment could not be analyzed in the same run. A Non Template Control (NTC) was included for each primer combination, to detect any potential contamination from gDNA and/or primer dimer (Leo et al., 2016).

### RT-qPCR Data Analysis

Cq data of all RGA that were differentially expressed between chickpea genotypes and treatments were imported into LinRegPCR software version 2017.1 (Ruijter et al., 2015) for further analyses. Samples that did not amplify or produced a low, high or inconsistent Cq value (under 5 or over 40 cycles) were removed. The raw Cq values of the expression of each RGA locus were then corrected according to their respective PCR efficiencies, and the mean values of the biological triplicates were calculated. The Delta-Delta-Cq (ddCq) algorithm was used to determine relative and differential expressions among varieties and treatments (Pfaffl, 2001). An R script was then used to generate the differential expression plots of each RGA locus. Relative expression data (ddCq) above 0 meant that the RGA gene at this time point/genotype was up-regulated in the treated compared to the control group, whereas negative ddCq indicated that the RGA gene was down-regulated at that point. A heatmap was constructed and displayed using R software based on the calculated mean fold-change in expression values among genotypes and time-points after normalization with the reference genes and untreated samples. Several statistical tests were then performed to provide evidence for real differences in RGA expression levels among genotypes and following inoculation: Firstly, a Levene test was performed to verify the homogeneity of variances, followed by a Shapiro–Wilk test to assess the normality of the variances. If both conditions were met, an ANOVA test was applied to compare the significance of expression differences between treated and untreated groups, otherwise, a non-parametric Kruskal–Wallis test was used to compare the groups. If the result was significant, pairwise comparisons among all sample groups were undertaken to test which group(s) were different from others using a Tukey test. All statistical analyses were carried out in the R Language and Environment for Statistical Computing (R Core Team, 2017). All R script developed for this study can be found at https://github.com/ziwei-zhou/Thesis_R_scripts. A *p*-value of 0.05 was used as the significance threshold in all statistical tests.

### Analysis of RGA Protein Sequences

Bioinformatics and predictive *in silico* tools were used to further characterize RGAs. The predicted amino acid sequence of each RGA candidate was obtained from the NCBI database and imported into InterPro 5^3^ (Jones et al., 2014) and KOBAS 3.0 software^4^ (Xie et al., 2011), which were used to classify the predicted proteins into families and to predict domains and important (i.e., binding) sites. The RGA that responded with the highest transcriptional response to the pathogen was chosen for secondary structure prediction using the Position Specific Iterated – BLAST based secondary structure prediction (PSIPRED) method^5^ (Jones, 1999). Three-dimensional atomic models of this RGA and its potential binding sites were predicted through RaptorX software^6^ (Källberg et al., 2012).

## Results

### RGA Locus Identification and Validation

In total, 25 RGA loci were identified from previous publications and based on known RGA motifs from within the chickpea sequences within the NCBI database. These were labeled from RGA 1 to 25. PCR products of the expected sizes were successfully amplified from 23 of the targeted putative loci across all four chickpea varieties assessed (Table 2). After primer efficiency testing, 10 RGAs produced a reliable and consistent linear amplification, based on their *R*^2^ result and E value (RGAs 4, 6, 8, 9, 10, 11, 12, 15, 21, and 23).

**Table 2:** PCR validation of RGA sequences.

RGA #	RGA LOCUS	Observed size (bp)	Source (citation or novel)
RGA 1	XM003599356.1	–	Palomino et al., 2009
RGA 2	DQ276889.1	–	Palomino et al., 2009
RGA 3	XM004512872.2	204∼208	Palomino et al., 2009
RGA 4	XM012712573.1	150∼155	Palomino et al., 2009
RGA 5	XM012713173.1	176∼182	Palomino et al., 2009
RGA 6	DQ276896.1	120∼125	Leo et al., 2016
RGA 7	AF186624.1	150∼155	Leo et al., 2016
RGA 8	DQ276915.1	245∼250	Leo et al., 2016
RGA 9	AJ307992.1	120∼125	Leo et al., 2016
RGA 10	KF460544.1	205∼210	Sagi et al., 2017
RGA 11	KF577584.1	195∼200	Sagi et al., 2017
RGA 12	KF571717.1	180∼185	Sagi et al., 2017
RGA 13	KF438082.1	160∼165	NCBI database
RGA 14	DQ276912.1	150∼155	NCBI database
RGA 15	DQ276896.1	200∼205	NCBI database
RGA 16	AJ307997.1	175∼180	NCBI database
RGA 17	AF186626.1	180∼185	NCBI database
RGA 18	AJ307986.1	250∼255	NCBI database
RGA 19	AJ307990.1	250∼255	NCBI database
RGA 20	XM004485780.2	230∼235	NCBI database
RGA 21	KF560326.1	218∼225	NCBI database
RGA 22	KF560323.1	225∼230	NCBI database
RGA 23	LOC101492873	112∼118	NCBI database
RGA 24	LOC101502375	72∼79	NCBI database
RGA 25	LOC101511908	110∼116	NCBI database

### Quantitative Real-Time Expression Profiling of the RGA Genes

**FIGURE 1:**
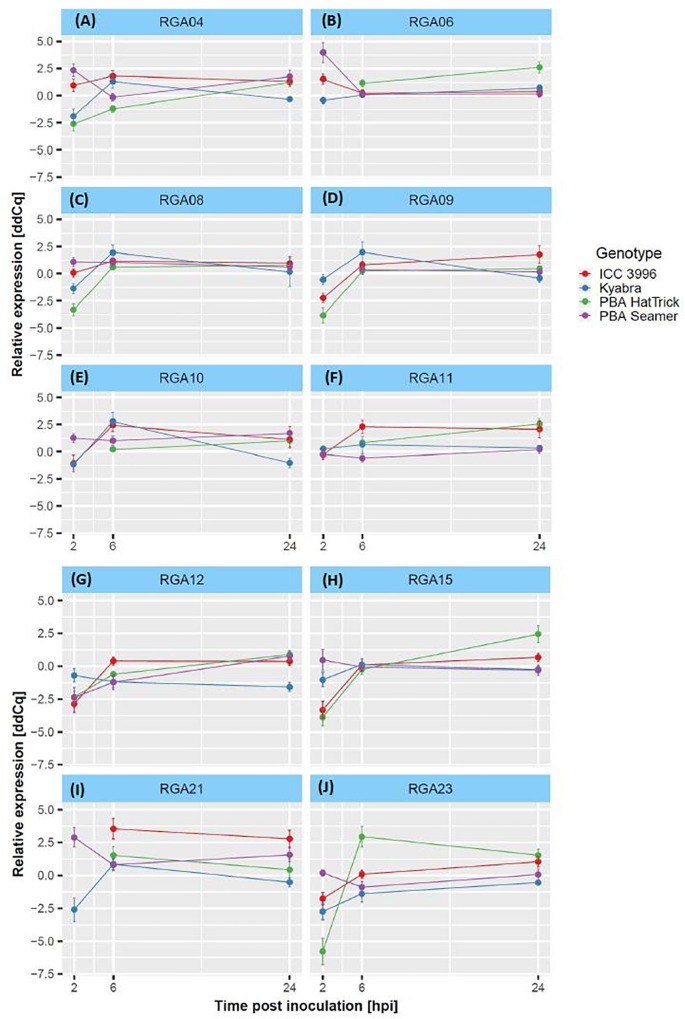
**(A–J)** Differential expression plots of 10 RGA loci among four chickpea genotypes (with reliable *R*^2^ and *E*-values) over the experimental time course after inoculation with the *A. rabiei* isolate, FT13092-1. Delta-Delta-Cq (ddCq) represents the relative expression ratio between treated and control samples, see Pfaffl (2001) and section “RT-qPCR Data Analysis” for details.

Differences in the transcription levels of the selected RGAs over time, after inoculation with isolate FT-13092-1, were observed among the four chickpea genotypes assessed (Figure 1A–J). Interestingly, RGA 8 and 10 were both significantly up-regulated at the earliest timepoint assessed, at 2 hpi and in only the resistant PBA Seamer and ICC3996 genotypes (Figure 1C,E). These then remained up-regulated for the duration of the experiment, potentially indicating their ability to recognize the pathogen prior to invasion. This may indicate that they provide sustained signaling, leading to the instigation of downstream defense occurring much faster in these genotypes than in the more susceptible ones. RGA 21 and 23 showed down regulations in ICC 3996 at the beginning of the experiment, and then sharply increased to up-regulations at 6 hpi (Figure 1I,J). Meanwhile, RGA 4, 9, and 15 were initially down-regulated with a subsequent sharp increase in most chickpea genotypes, potentially indicating an overall ability of these RGA to recognize the pathogen following invasion, possibly too late for effective defense signaling (Figure 1A,D,H). While the expression profiles of RGA 6, 11, and 12 were not so significant in the plots (Figure 1B,F,G).

**FIGURE 2:**
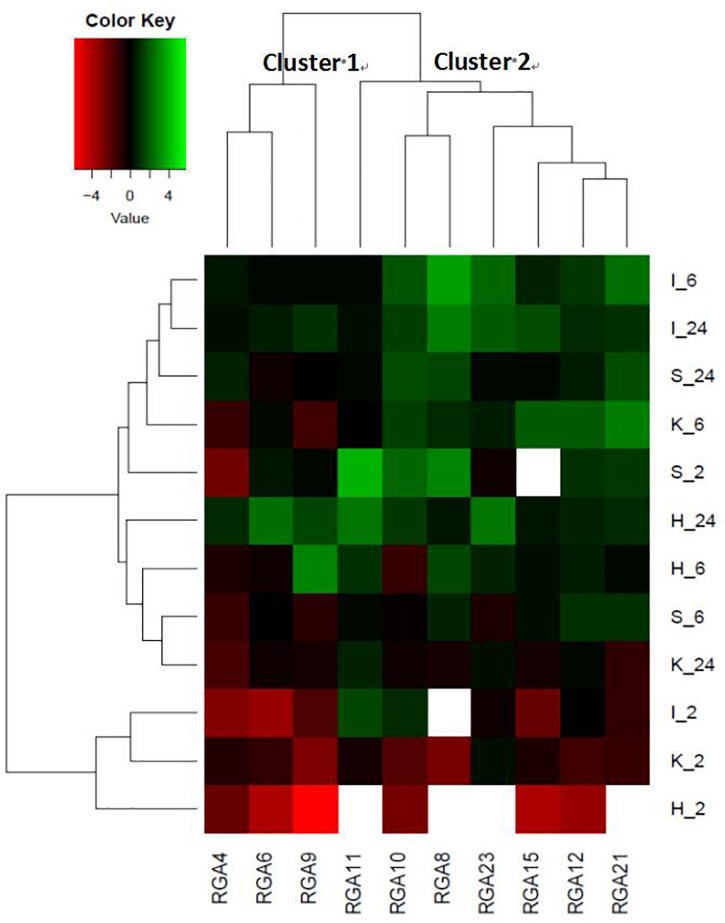
A heatmap representing the fold-change differences in expression among the 10 RGA target loci at 2, 6, and 24 hpi in four chickpea cultivars (PBA Seamer, PBA HatTrick, Kyabra, ICC 3996; so I_6 = ICC 3996_6hpi, same as others). Green color represents up-regulation, black color represents no change and red color represents down-regulation and color intensity indicates fold-change. No detectable expression is represented in white. The mean fold change expression values of the expression profiles for each treatment and genotype were normalized with the two mentioned reference genes and untreated samples.

The relationships among the differential mean fold-changes of expressions of the 10 RGAs during the time-course were observed in the heatmap (Figure 2). Cluster 1 comprised of RGAs 4, 6, and 9. These were either down-regulated or unchanged for all genotypes (except in PBA HatTrick) at all time points assessed. Cluster 2 comprised of RGAs 8, 10, 21, and 23. These were up-regulated at 6 and 24 hpi and as stated above, RGA 8 and 10 were also up-regulated at 2 hpi in ICC3996, the commonly used *A. rabiei* resistance source in the Australian breeding program (Mehmood et al., 2017).

### Prediction of RGA Functional Groups

**FIGURE 3:**
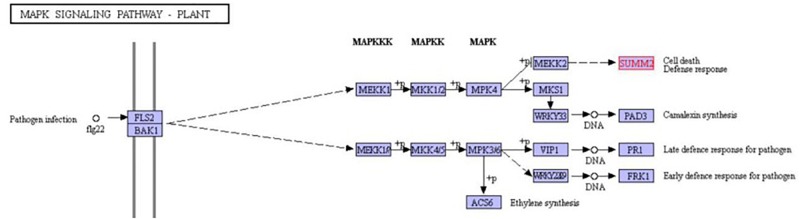
Part of the plant MAPK signaling pathway. The location of gene SUMM2 is labeled in red block (Kanehisa and Goto, 2000).

RGAs 8, 10, 21, and 23 were further assessed through *in silico* analyses to predict functional involvement in *A. rabiei* recognition. Their homologous super families and amino acid sequences were predicted (Table 3 and Supplementary Table 2, respectively) and NCBI reference sequences (RefSeq), gene and protein IDs were retrieved (Table 3). Domains and motifs were also predicted (Table 4). Whilst none of the four interrogated RGAs were able to be fully annotated, potentially indicating novelty, all were highly homologous (90–99% identity) with SUMM2 (KEGG orthology number K20599; Zhang et al., 2012). SUMM2 is an NB-LRR protein known to function in plant mitogen-activated protein kinase (MAPK) signaling pathways (Figure 3).

**Table 3:** Homologous super family predictions of the four chickpea target RGA sequences and their reference sequences definitions.

RGA loci	RefSeq Gene ID/ Protein ID	RefSeq definition	Homologous super family
RGA 8	101493284/ XP_004492464	TMV resistance protein N-like	P-loop containing nucleoside triphosphate hydrolase
RGA 10	101502658/ XP_004499578	Putative disease resistance RPP13-like protein 1	P-loop containing nucleoside triphosphate hydrolase; Leucine-rich repeat domain superfamily
RGA 21	101504229/ XP_012568623	Putative disease resistance protein At3g14460	P-loop containing nucleoside triphosphate hydrolase
RGA 23	101492873/ XP_004498272	uncharacterized LOC101492873	P-loop containing nucleoside triphosphate hydrolase; Leucine-rich repeat domain superfamily

**Table 4:** Protein sequence analyses and characterizations of the four chickpea target RGA sequences.

RGA gene	Domain description	Motif id	Start	End	Length (aa)
RGA 8	AAA ATPase domain	pf:AAA_16	213	329	116
RGA 8	NB-ARC domain	pf:NB-ARC	223	469	246
RGA 8	AAA domain	pf:AAA_14	232	332	100
RGA 8	NACHT domain	pf:NACHT	233	379	146
RGA 8	AAA domain	pf:AAA_22	235	334	99
RGA 10	Arabidopsis broad-spectrum mildew resistance protein RPW8	pf:RPW8	4	88	84
RGA 10	Putative tranposon-transfer assisting protein	pf:TTRAP	40	82	42
RGA 10	AAA ATPase domain	pf:AAA_16	171	282	111
RGA 10	NB-ARC domain	pf:NB-ARC	175	455	280
RGA 10	ATPase domain predominantly from Archaea	pf:ATPase_2	176	295	119
RGA 10	AAA domain	pf:AAA_22	196	283	87
RGA 10	NACHT domain	pf:NACHT	197	342	145
RGA 10	AAA domain	pf:AAA_14	197	312	115
RGA 10	AAA domain	pf:AAA_33	197	297	100
RGA 10	AAA domain	pf:AAA_18	198	290	92
RGA 10	Leucine rich repeats (2 copies)	pf:LRR_4	581	620	39
RGA 10	Leucine rich repeat	pf:LRR_8	581	637	56
RGA 10	Leucine rich repeats (2 copies)	pf:LRR_4	606	643	37
RGA 10	Leucine rich repeats (2 copies)	pf:LRR_4	628	663	35
RGA 10	Leucine rich repeat	pf:LRR_8	646	680	34
RGA 10	Leucine rich repeats (2 copies)	pf:LRR_4	652	681	29
RGA 10	Leucine rich repeats (2 copies)	pf:LRR_4	788	817	29
RGA 21	AAA ATPase domain	pf:AAA_16	168	256	88
RGA 21	NB-ARC domain	pf:NB-ARC	191	456	265
RGA 21	AAA domain	pf:AAA_14	196	308	112
RGA 21	AAA domain	pf:AAA_22	197	281	84
RGA 21	AAA domain	pf:AAA_18	198	289	91
RGA 21	Leucine rich repeat	pf:LRR_8	595	633	38
RGA 21	Leucine rich repeats (2 copies)	pf:LRR_4	604	642	38
RGA 21	Leucine rich repeat	pf:LRR_8	626	678	52
RGA 21	Leucine rich repeats (2 copies)	pf:LRR_4	787	825	38
RGA 23	ArgK protein	pf:ArgK	147	191	44
RGA 23	PhoH-like protein	pf:PhoH	148	201	53
RGA 23	ATPase domain predominantly from Archaea	pf:ATPase_2	149	234	85
RGA 23	AAA ATPase domain	pf:AAA_16	150	200	50
RGA 23	NB-ARC domain	pf:NB-ARC	154	415	261
RGA 23	AAA domain	pf:AAA_30	154	231	77
RGA 23	AAA domain	pf:AAA_22	166	253	87
RGA 23	NACHT domain	pf:NACHT	168	253	85

*aa, Predicted amino acid sequence length.*

**FIGURE 4:**
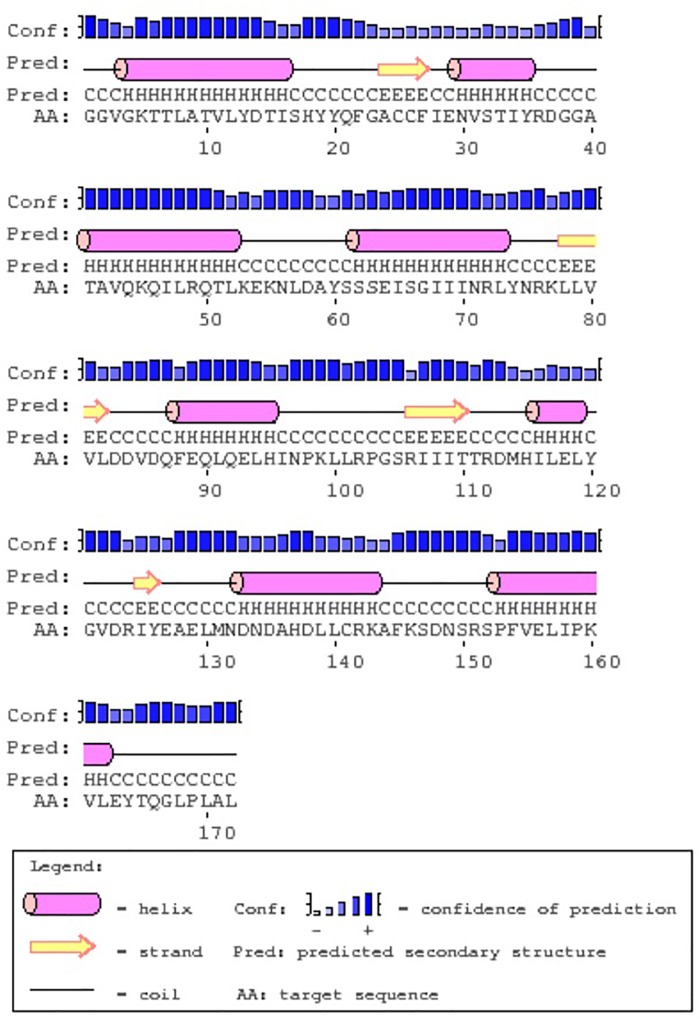
The predicted secondary protein structure derived from the PSIPRED server for the chickpea RGA 8.

**FIGURE 5:**
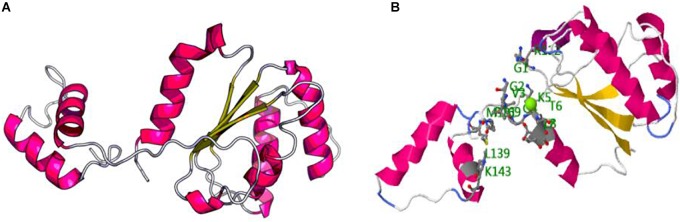
The predicted RGA 8 3D protein structure **(A)** and the proposed binding site residues (**B**, the red, blue, and green balls in the right picture).

RGA 8 responded with the highest and earliest transcriptional response to the pathogen and so was chosen for further secondary structure prediction that revealed eight α-helices and four β-strands (Figure 4). The top predicted binding site domains for potential external sequences were identified with predicted binding residues at positions G1, G2, V3, G4, K5, T6, T7, L8, R112, M131, L139, K143, P169, and L170, and their collective predicted ligands were Magnesium ion (Mg^2+^), Adenosine diphosphate (ADP) and exchanging adenosine triphosphate (ATP) (Figure 5).

## Discussion

Plants have their own effective innate immune systems that they use to recognize pathogens when they come into contact or begin to invade and cause infection (Höhl et al., 1990; Ilarslan and Dolar, 2002; Jayakumar et al., 2005). Most necrotrophic pathogen-plant pathosystems utilize *R*-gene families otherwise known as RGAs as the receptors for initial pathogen perception (Sekhwal et al., 2015). For the chickpea-*A. rabiei* pathosystem, this study has assessed several existing and newly identified RGAs for their involvement in this perception process, which is proposed to lead to downstream signaling of biochemical and physical defense mechanisms (Palomino et al., 2009; Leo et al., 2016; Mehmood et al., 2017; Sagi et al., 2017).

The timing of RGA expression is thus crucial for a plant to be able to recognize a pathogen fast enough to incite effective defense responses. In this study, we found that a cluster of RGAs (Cluster 2), was up-regulated by 2–6 h following inoculation with a highly aggressive *A. rabiei* isolate and that this was consistent with the timing of spore growth (germ tube elongation) and penetration (appressoria development) (Sambasivam et al., 2008). If a plant can recognize and initiate defense responses faster, it may be able to contain the fungus long enough for more systemic resistance responses to occur, including hormone signaling, structural rearrangement and production of pathogenesis proteins. These alert the whole plant to the presence of the pathogen and direct a concerted attack at the site of invasion. This was proposed to be the case in the lentil – *Ascochyta lentis* pathosystem, whereby the host genotype was able to recognize and defend itself against the pathogen faster and was able to incite production of toxic phenolic compounds in a hypersensitive response as well as strengthen the cell wall around the invading hyphae compared to the slower and susceptible genotype (Sambasivam et al., 2017; Khorramdelazad et al., 2018). The fast recognition of the pathogen by several RGAs assessed in the chickpea – *A. rabiei* pathosystem stands in agreement with the observation of Leo et al. (2016) and Sagi et al. (2017) who also observed up-regulation as early as 2–6 hpi.

Since ICC3996 is the most widely used resistance source in breeding new resistant chickpea cultivars in Australia (Mehmood et al., 2017), it was important to determine which of the responsive RGA are present in this genetic background. In Cluster 2 of the heatmap (Figure 2), RGA 8, 10, 21, and 23 were up-regulated at 2–24 hpi in ICC3996. The homologous super family predictions indicated a common evolutionary origin among these four RGAs as evidence by the nucleoside triphosphate hydrolase domain (P-loop NTPase) (Leipe et al., 2003). P-loop NTPase is the most prevalent nucleotide-binding protein domain, catalyzing the hydrolysis of the beta-gamma phosphate bond of a bound nucleoside triphosphate (NTP) (Arya and Acharya, 2017). It is possible that these responsive RGAs in chickpea are Signal Transduction ATPases with Numerous Domains (STAND) P-loop NTPases and may function by ATP to initiate effector-triggered immunity (ETI) signaling.

RGA 8 was up-regulated in ICC3996, PBA Seamer, and PBA HatTrick at all times assessed, indicating that this locus is robust in its response to the pathogen. Also, since PBA Seamer and PBA HatTrick are progeny of crosses containing ICC3996 as the resistance donor parent (Dr. Kristy Hobson, Australian Chickpea Breeder, pers. comm.), this highlights that RGA 8 is heritable and may be selected for as a major contributor to the resistance response. The region containing the “GGVGK” domain in RGA 8 was proposed as a magnesium ion binding site, believed to induce phospho-transfer reactions (Li et al., 2001). This region was once showed resistance in tobacco after tobacco mosaic virus (TMV) infections (Les Erickson et al., 1999), and in response to *Synchytrium endobioticum* in potato (Hehl et al., 1999). Further, as mentioned, the secondary structure prediction for the RGA 8 revealed eight α-helices and four β-sheets (Figure 4), which is similar to the predicted plant disease resistance gene product reported by Rigden et al. (2000), found to function in His-Asp phosphor-transfer pathways. Therefore, the function of RGA 8 within defense to *A. rabiei* in chickpea may logically be predicted as a receptor to trigger the phospho-transfer signaling pathway through the activation of MAPK cascades.

Interestingly, RGA 10 was up-regulated in ICC 3996 and PBA Seamer but not in PBA HatTrick. The “resistant” status of PBA HatTrick was revised from “moderately resistant” to “moderately susceptible” in February 2017 by Pulse Breeding Australia, due to a substantial increase in aggressiveness within the isolate population (Mehmood et al., 2017). Meanwhile both ICC 3996 and PBA Seamer remained “resistant” at the time. RGA 10 contains domains homologous to Arabidopsis broad-spectrum mildew resistance protein RPW8 and a putative transposon-transfer assisting protein (TTRAP) (Xiao et al., 2001; Pulavarti et al., 2013). RPW8 is involved in resistance to a broad range of powdery mildew pathogens and TTRAP is associated with a family of small bacterial proteins largely derived from *Clostrium difficile* (Pulavarti et al., 2013). One could postulate that the functionality of the chickpea RGA 10 may have been lost in PBA HatTrick when exposed to a new highly aggressive isolate such as the one used in this study. This highlights the evolutionary risk of relying on one or few RGA (*R*-genes) for sustained resistance, as has been proven over again in other crops such as cereals in the race to breed for resistance against rust pathogens and in canola against the blackleg pathogen (Burdon et al., 2014; Moore et al., 2015a; Periyannan et al., 2017; Bousset et al., 2018; Zhang and Fernando, 2018).

RGA 21 was also up-regulated at 6 and 24 hpi in ICC 3996, meanwhile, it showed up-regulation in the susceptible Kyabra at 6 hpi. As showed in Table 4, RGA 21 contains a NB-ARC domain, a AAA ATPase domain, and a Leucine Rich Repeat, all belonging to the NBS-LRR family. Meanwhile, RGA 23 contained homologs of ArgK and PhoH-like proteins. ArgK is a member of the of P-loop GTPases, involved in the transport of positively charged amino acids (lysine, arginine, and ornithine) and has arginine kinase activity (Leipe et al., 2002). Previously, this was only found to exist in eukaryotic *Caenorhabditis* and *Leishmania* species. Similarly, the PhoH-like protein is a cytoplasmic protein. which has been shown to act in phosphate regulation in *Escherichia coli* (Kim et al., 1993). Further analyses will determine if the chickpea genes are complete and potentially functional.

Finally, the predicted proteins of all four RGAs share high similarities with the NB-LRR protein SUMM2 (Figure 3). SUMM2 is proposed to be activated with the MEKK1-MKK1/MKK2-MPK4 cascade when the MAPK signaling pathway is disrupted by pathogen effector binding, leading to the responses that cause localized cell death (Zhang et al., 2012). This indicates the potential for these RGA candidates to activate the well characterized defense responses to *A. rabiei* in chickpea when the MAPK signaling pathway is potentially suppressed by *A. rabiei* leading to apoptosis and the observed hypersensitive response (Leo et al., 2016; Mehmood et al., 2017).

## Conclusion

Although many studies have been devoted to improving chickpea resistance to *A. rabiei*, sustained success may in part have been limited due to a lack of accurate knowledge of the pathogen recognition mechanism and how it may lead to subsequent instigated defense mechanisms. This is despite a great deal of effort in genetic mapping and characterization of multiple contributory defense-related QTLs, and their identification in diverse genetic backgrounds (Coram and Pang, 2005a,b; Palomino et al., 2009; Sagi et al., 2017). Although the physical locations of several genes underpinning the resistance responses have been uncovered, few studies have contributed to discovering the structures and functions of the actual resistance proteins. Fortunately, a great deal of knowledge exists on resistance proteins structure and function, as well as the molecular mechanisms of defense signaling proteins in Solanaceous plants (summarized by van Ooijen et al., 2007), which provides a guiding model for exploring the classes and functions of resistance proteins in other plant species. In this research, several existing and newly identified RGAs in chickpea were classified into previously described classes and assessed for their involvement in the *A. rabiei* perception process, which is proposed to lead to downstream signaling of biochemical and physical defense mechanisms (Palomino et al., 2009; Leo et al., 2016; Sagi et al., 2017). In conclusion, the future directions of this study should be focused on unraveling the protein functions of the selected RGAs that were differentially expressed in the resistant chickpea varieties after *A. rabiei* infection. This will provide further evidence for the selection of key RGAs in resistance breeding approaches.

## Author Contributions

ZZ designed and conducted the experiments, analyzed the data, and wrote the manuscript. RF directed the project, co-designed the experiments, and edited the manuscript. IB and PS assisted with the experiments and data analyses.

## Conflict of Interest Statement

The authors declare that the research was conducted in the absence of any commercial or financial relationships that could be construed as a potential conflict of interest.

## Footnotes

1 https://www.ncbi.nlm.nih.gov

2 http://bioinfo.ut.ee/primer3-0.4.0/

3 https://www.ebi.ac.uk/interpro/

4 http://kobas.cbi.pku.edu.cn/annotate.php

5 http://bioinf.cs.ucl.ac.uk/psipred/

6 http://raptorx.uchicago.edu/
