# Supplementary material for: Determination of the Key Resistance Gene Analogs Involved in Ascochyta rabiei Recognition in Chickpea
Source: Front Plant Sci. 2019 May 17;10:644. doi: 10.3389/fpls.2019.00644 (PMC6546118; doi:10.3389/fpls.2019.00644)
Supplement: Supplementary file 1 [file Table_1.DOCX]

Supplementary Material

Determination of the key resistance gene analogues involved in *Ascochyta rabiei* recognition in chickpea

Ziwei Zhou, Ido Bar, Prabhakaran Thanjavur Sambasivam, Rebecca Ford*

Environmental Futures Research Institute, School of Environment and Science, Griffith University, Nathan campus, Queensland, Australia 4210.

***Correspondence:**Rebecca Ford
Rebecca.ford@griffith.edu.au

**Supplementary Table 1**. Sequences of specific primers and identifications based on RGA sequences from published papers and NCBI

| RGA # | RGA LOCUS | Forward primer seq | Reverse primer seq | Description | Reference |
| --- | --- | --- | --- | --- | --- |
| RGA 1 | XM003599356.1 | CGGTCACTTCAAGAACATGG | GTGCATGCTACCTTAGCAAC | Medicago truncatula LRR and NB-ARC domain disease resistance protein partial mRNA | Palomino et al. (2009) |
| RGA 2 | DQ276889.1 | TCAAAGGTATGGGAAAGCGAT | TGTCTTCGCCATCTCTACAA | Vicia faba clone AVI1.14 NBS-LRR type disease resistance protein gene, partial cds |  |
| RGA 3 | XM004512872.2 | GCTGGTGACTCCTTTCGTTG | CCAATCGCTTCTAGGCTTGC | PREDICTED: Cicer arietinum putative disease resistance RPP13-like protein 1 (LOC101501665), transcript variant X1, mRNA |  |
| RGA 4 | XM012712573.1 | ACAGAAATTCAATGTTCAAGCC | TTCCGGTCAGCTTATCCTTTAAC | PREDICTED: Cicer arietinum putative disease resistance protein At3g14460 (LOC101505696), mRNA |  |
| RGA 5 | XM012713173.1 | GATGTTCAAGCATGGGTCTG | TCGTTCCAGACATCATCCAG | PREDICTED: Cicer arietinum putative disease resistance protein At3g14460 (LOC101504229), transcript variant X5, mRNA |  |
| RGA 6 | DQ276896.1 | GGCCATTGAATCAAGACGAG | CACATTTCACCACAATCTCC | Cicer arietinum clone FVI1.18 NBS-LRR type disease resistance protein gene, partial cds | Leo et al. (2016) |
| RGA 7 | AF186624.1 | GAACGACGACCAAGATAC | CCATTTACGACTTCCGCAC | Cicer arietinum clone CP2 unknown gene |  |
| RGA 8 | DQ276915.1 | GCGACCGTCTTGTATGACAC | GGAGCTTCCTGTTGTATAGCC | Cicer arietinum clone FII1.6 NBS-LRR type disease resistance protein gene, partial cds |  |
| RGA 9 | AJ307992.1 | TGCCGTATTGCTGATCTGA | TAGATGCGTTGTGAAGATT | Cicer arietinum partial rga-G gene |  |
| RGA 10 | KF460544.1 | GTTGCACACACATTCCCCAT | TTGAACGCAAAAGTCCTCCG | PREDICTED: Cicer arietinum putative disease resistance RPP13-like protein 1 (LOC101493845), mRNA | NCBI with gene accession number |
| RGA 11 | KF577584.1 | ACCGTGAATGTTTTGGTGCA | CTTACGTGCCAACCGTTCAT | Cicer arietinum isolate Ca_09445 NBS-LRR protein gene, complete cds |  |
| RGA 12 | KF571717.1 | CTTGAAGTTTCTGCGGGCTT | CGGCACCCAGTCAATTTCAA | Cicer arietinum isolate Ca_12872 NBS-LRR protein gene, complete cds |  |
| RGA 13 | KF438082.1 | GGTGCTTTGGTTGATGGTGA | AGATAGTAAGCCTCCCACGC | Cicer arietinum clone Ca8 NBS-LRR protein gene, complete cds |  |
| RGA 14 | DQ276912.1 | GGAAGACGACCCTAGCTCAA | TGCTTTCCCAAACACTCGAA | Cicer arietinum clone FVI1.13 NBS-LRR type disease resistance protein gene, partial cds |  |
| RGA 15 | DQ276896.1 | TGGGCTATGGAAGTTTGGGA | CGGTATCACATTTGCTCCCG | Cicer arietinum clone FVI1.18 NBS-LRR type disease resistance protein gene, partial cds |  |
| RGA 16 | AJ307997.1 | TGGATGACGTTTTGGATGCA | CCCGCAATCTTCACTTCCAA | Cicer reticulatum partial rga-F3 gene |  |
| RGA 17 | AF186626.1 | GTTGGGCAGGTGTCTTTACA | GACGACTTCCACACAAAGCA | Cicer arietinum clone CP4 unknown gene |  |
| RGA 18 | AJ307986.1 | TCAACTCACTTGCTTTCTTGC | TTTGCTTCCTGGACCAAACC | Cicer arietinum partial rga-A gene |  |
| RGA 19 | AJ307990.1 | GTCTACGAAGCGATCAAAGATG | TCCAAGCTAAACTCTCCAATTGG | Cicer arietinum partial rga-E gene |  |
| RGA 20 | XM004485780.2 | TCTCGACAGGATGAGGCTTC | TACAATTGGAATGCGCGAGG | PREDICTED: Cicer arietinum pentatricopeptide repeat-containing protein At5g46100 (LOC101510733), mRNA |  |
| RGA 21 | KF560326.1 | CCCAAGCTGTGTACAACCAT | CGGTCCAGACATCATCCAGA | Cicer arietinum clone Ca6 CC-NBS-LRR disease resistance protein gene, complete cds |  |
| RGA 22 | KF560323.1 | GGATGCGGTAAAAGACGCTT | CCCGCCACAACTTTTAACCA | Cicer arietinum clone Ca3 CC-NBS-LRR disease resistance protein gene, complete cds |  |
| RGA 23 | LOC101492873 | CCTAAGACAGAGAATGCCACAA | CAACTGTGGTGACTGTGAAGA | PREDICTED: Cicer arietinum uncharacterized LOC101492873, transcript variant X2, mRNA | Sagi et al. (2017) |
| RGA 24 | LOC101502375 | TGATATGCACAAGGTGGATGTAG | CACCTGAGAAGACGGCATAAA | PREDICTED: Cicer arietinum uncharacterized LOC101502375, mRNA |  |
| RGA 25 | LOC101511908 | GGGCAGTGCCTCTGATGTAT | GCTTAGCTCTGCAACTGGCT | PREDICTED: Cicer arietinum uncharacterized LOC101511908, transcript variant X1, mRNA |  |

**Supplementary Table 2.** The predicted amino acid sequences and the annotated reference sequence (RefSeq) of RGA 8, RGA 10, RGA 21 and RGA 23.

| RGA Locus | Amino acid sequence |
| --- | --- |
| RGA 8 | GGVGKTTLATVLYDTISHYYQFGACCFIENVSTIYRDGGATAVQKQILRQTLKEKNLDAYSSSEISGIIINRLYNRKLLVVLDDVDQFEQLQELHINPKLLRPGSRIIITTRDMHILELYGVDRIYEAELMNDNDAHDLLCRKAFKSDNSRSPFVELIPKVLEYTQGLPLAL |
| RGA 8 RefSeq | MSKRTFGDIIGEGGSSSSSSKEFDSIQSYRYIYDVFISFRGVDTRNTFVDHLYAHLIRKGIFVFKDDKQLHKGNSISRQLLQAIQHSRISIIVFSKDYASSTWCLDEMAAIAECHRELKQPVFPIFYDVDPSCVRKQIGLYESAFLLLAEQFKHDPHKVDGWKRAMTCLGGSAGWDVRNKLEFEEIEKIVQAVIKTLGHKFSGFADDLIGMQPRVEALKSILRLSSEDDGFRVLGIWGMGGIGKTTLATVLYDTISHYYQFGACCFIENVSTIYRDGGATAVQKQILRQTLKEKNLDAYSSSEISGIIINRLYNRKLLVVLDDVDQFEQLQELHINPKLLRPGSRIIITTRDMHILELYGVDRIYEAELMNDNDAHDLLCRKAFKSDNSRSPFVELIPKVLKYTQGLPLAIRIMGSFLFNRNAMQWRATFDGLQNNPDKRIMKVLQMSFEGLQLREREIFLHVACFFEGEREDYVRRILHACGLQPNIGIPLIAEKSLITIRNQVICMHKMLLELGKQIVQGQHLDEPRFWNRLWLYRDFHHVMMTKVEATETKAIVLDQKEDGFKFSKLRVEDLSKMEHLKLLILNHKNFSGKPIFLSNSLYYFSWNGYPFTALPSNFQPYDLVELNMPDSNIEQLWEGIQCLPCLKRMDLSNSKNLKMTPCFDGILNLERLDLTGCINLLQVHPSIGLLSELVFLSLQKCSSLVHLDFGSASRLWSLRVLLLSGCTKLKSTPDFTGAINLEYLDVDHCASLFTFDGSLAKLRVLSLRDCTNLVRLGDSLDKMTSLTTLDLCGCLKYKCLPVNSAMKSLVFLDLSFCNISNLPQAIGELRGLERLNLQGNNFTAIPSLQCLSNLAYLNLSYCHCLQRLPQLPTTSGPSDSMGIYFKTTSGSRDHRSGLYVFDCRKIFGGSIWISSIDHQYEWLQRLLKEPRHFRCGFDIVLPFPCYGSAEIPMWFDHRYKGGSIIRIKNFDVNVDWVGFVFFVQFEVRYPMPNSASSQHPYPFYLSFESEHTEERFDMPLSLKSNKVDGSPSEYCWTIYISQEHCHFVKTEAQITFKAYQGLIMKEWGFHMLTEKERKYVDQSNMKKVCLWSQTVSSCLV |
| RGA 10 | MAATLVGGAFLSATVQTLVEKLASTEFRDYIKNTKLNESLLAELKTTLLALQAVLDDAEMKQITNTAVETWLDELKDAIFDAEDLLNQINYDALRCKMEVKQGENITGQVWNLISSPFKNIYAEINLQMKNMCQRLQLFAQQRNILGLQTVSGRVSRRTPSSSMVNESFMVGRKDDKEELISMLLSHSDTNSNNIGVVAIWGMGGVGKTTLAQLLYNDKKVQEHFDLKVWVCVSEDFNILRVTKSLLESITSRGSESTNLDFLRRELNKNLSDKRFLLVLDDLWNDNYNDWDELVTPLVNGKIGSRVIITTRHQKVAEVAHTFPIHKVETLSDDDCWSLLSKHAFRSENCQSNESPNLEAIGKKIAKKCGGLPIAAKTLGGLLRSKVDAKEWAEILNSDIWKLTKDNILPALRLSYQDLPSHLKRCFAYCSIFPKDFPFDRKQLVLLWMAEGFLDHSLENKTPEEVGDDYVNELLSRSLIQVQQSNGNFGRQKLVMHDLVNDLASMVSGKSYFRLECSSKISKNVRHLSYNQEFYDIFKKFEVLDNFKCLRSFLPVNLFDFSYDYYLSRKVVEDLLPKLIRLRVLSLSSYRNITTLPESIGRMVLLQYLDLSFTGIKSLPDAICKLYNLQTLNLMGCMDLTELPLCFGQLINLRHLDISETNINEMPKQIVALENLQTLTVFVVGKQEIGLSVKDLGKFPNLQGDLCIKNLHNVIDAVDASDANMRSKKHIEELELQWSKQTEDSRMEKDVLDMLKPSLNLKKLSIDLYGGTSFPSWLGDSSFSNMVSLCISNCEYCITVPPLGQLPSLKDLTISGMKMEIIGIEFYGMTLEPSISSFQPFRSLEKLNFDCMPNWKEWIHYESGEVAFPRLRTLSLSKCPKLMGHMPNHLSSIDEVSITCCDSLLTTPHTTLHWVSSLKKISIQKDQNSLMVSKRTQLLLPVSERTQWLLPENDSPCLLQSLFIRYYDTLFSIPKIIMSSNCLQSLKLYDIPSLGAFPTDGLPTSLQSLTIYNCENLAFLPLETWGNYTSLVNLHLAKSCNSLTSFALDGFPTLQNLHIDSCTNLESIFISKTSLLLPSSLTPRMETLTSLEYLYLKNLPKLSFCEGACLPPNLGCIYIGSVRITTPIFEWGLQRLTALSTLSIGSDDDIVNTLLKEKLLPISLRDLFIHHLSEIKSFEGNGLRHLSSLEALCFNDCPRLEYLLEDTLPSSLKILRIVKCPLLEARYKSQRLEHLSNLVLRINGEVII |
| RGA 10 RefSeq | MAATLVGGAFLSATVQTLVEKLASTEFRDYIKNTKLNESLLAELKTTLLALQAVLDDAEMKQITNTAVETWLDELKDAIFDAEDLLNQINYDALRCKMEVKQGENITGQVWNLISSPFKNIYAEINLQMKNMCQRLQLFAQQRNILGLQTVSGRVSRRTPSSSMVNESFMVGRKDDKEELISMLLSHSDTNSNNIGVVAIWGMGGVGKTTLAQLLYNDKKVQEHFDLKVWVCVSEDFNILRVTKSLLESITSRGSESTNLDFLRRELNKNLSDKRFLLVLDDLWNDNYNDWDELVTPLVNGKIGSRVIITTRHQKVAEVAHTFPIHKVETLSDDDCWSLLSKHAFRSENCQSNESPNLEAIGKKIAKKCGGLPIAAKTLGGLLRSKVDAKEWAEILNSDIWKLTKDNILPALRLSYQDLPSHLKRCFAYCSIFPKDFPFDRKQLVLLWMAEGFLDHSLENKTPEEVGDDYVNELLSRSLIQVQQSNGNFGRQKLVMHDLVNDLASMVSGKSYFRLECSSKISKNVRHLSYNQEFYDIFKKFEVLDNFKCLRSFLPVNLFDFSYDYYLSRKVVEDLLPKLIRLRVLSLSSYRNITTLPESIGRMVLLQYLDLSFTGIKSLPDAICKLYNLQTLNLMGCMDLTELPLCFGQLINLRHLDISETNINEMPKQIVALENLQTLTVFVVGKQEIGLSVKDLGKFPNLQGDLCIKNLHNVIDAVDASDANMRSKKHIEELELQWSKQTEDSRMEKDVLDMLKPSLNLKKLSIDLYGGTSFPSWLGDSSFSNMVSLCISNCEYCITVPPLGQLPSLKDLTISGMKMEIIGIEFYGMTLEPSISSFQPFRSLEKLNFDCMPNWKEWIHYESGEVAFPRLRTLSLSKCPKLMGHMPNHLSSIDEVSITCCDSLLTTPHTTLHWVSSLKKISIQKDQNSLMVSKRTQLLLPVSERTQWLLPENDSPCLLQSLFIRYYDTLFSIPKIIMSSNCLQSLKLYDIPSLGAFPTDGLPTSLQSLTIYNCENLAFLPLETWGNYTSLVNLHLAKSCNSLTSFALDGFPTLQNLHIDSCTNLESIFISKTSLLLPSSLTPRMETLTSLEYLYLKNLPKLSFCEGACLPPNLGCIYIGSVRITTPIFEWGLQRLTALSTLSIGSDDDIVNTLLKEKLLPISLRDLFIHHLSEIKSFEGNGLRHLSSLEALCFNDCPRLEYLLEDTLPSSLKILRIVKCPLLEARYKSQRLEHLSNLVLRINGEVII |
| RGA 21 | MAKVVGEAPLSYLIVVLLDRLSSPQVANFIIGKKLDVNLIQRLKNTLYAVEAVLNDAEYKQIKDSAVANWLHDLIDALYLAYDILDLISTKEHLADDILNSTTASTYLCHFSNSEEMDMVRKLEYIVTRFESILKRKDILGLQHIATHHHSSWRTPSTSLNHGSKIFVDMGGVGKTTLAQAVYNHENIKQKFDVQAWVCVYDDFDVLKVTKAIAEEVRSACNTNNLNILQQDLKDKLSGKKFLIVLDDVWTEDYDSWNSLIKPLRCGAKGSKILVTTRIQKVVSMVQTFQAYSLEQLSDEDCWSVFANHTCLSQEKSTQNMDLEKIGKEIVRKCKGLPLAAQSLGGLLRRHDIRD |
| RGA 21 RefSeq | MDAAALGGAFLSAFIEVVLDRLASPQFANFITGNKLDVNLIQRLKNTLYAVEAVLNDAEQKQIKDSAVNNWLHDLKDALYIADDLLDHISTKSATTLKNKEVNTTATNYLSSFFNFEERDMVRKLEVIVARLESILKLKDILNLQHIATHHHSSWRTPSTSLDHGSNIFGRNQDIEAILKLLLHDGDDGDSDKISVIPIVGMGGVGKTTLAQSLYNHDTIKKKFDVQAWVCVSDDFDVLKVTKAIAEEVRSACNTNNLNILQQDLKDKLTGKMFLIVLDDVWNEDYDSWNSLIKPLRCGAKGSKILVTTRIQKVVSMVQTFQGYYSLEQLSNDDCWSVFANHACLSPEESTQNMDLEKIGKEIVRKCKGLPLAAQSLGGLLRRKRDIRDWNNILNSNIWEIDEKESKIIPALRISYHYLPPYLKRCFVYCSLYPKDYEFHADDLILLWMAEDLLQPPKNGKTLEEVGYEYFNDLASRSFFQRFVSGNSSRRFVMHDLVHDLATLVGAEFYFRTQELGKETKIGNKTRHLSFSNLSNRVTDNFEIFDQVKHLRTFLTIDFRRTPFNNENASCIILSNLKCVRVLSFQGFRDLNALPDSIGELIYLRYLDLSSTNIKTLPESLCNLHNLQTFKLYNCYQLTKLPNGMQKLVNLRYLDIRFTLKLEDMPREMSKLNHLQHLSCFVVGKHKEKGIKELGTLSNLHRSLSISKLENVSNNFEASQAKIMDKKYLEKLSFEWSDNAKDQFKNSQSEMDILDKLQPAKNLKKLSICGYRGTRFPEWVGDPSYRNLTKLSLSDCDNCCILPPLGQLQNLKDLRISRMSMLETIGYEYGDSFSGIIFPSLERLEFDNMPCWEVWHHPHYSNAYFPVLKSLVISGCPLLRGDLPSHFPALKTIQIEQCNQLASSLPYAPAIRKIEICESNKVALRELPLSLEEIKIDGREATESFFEIIAITLPISLKIIKINNCSCALSFPGDCLPASLLKLSIINCKNIDFLKQKQQHESLQWLYIDSSCDSLTIFPLETFPNLGSLHIHKCENLEFLSASKTLQNLNELFITKCPKLVSFARDGLAAPNLRSLVVCRCVNLKSLPCHANTLLPNLEMVRIWDCPEMETFCEGGMPPSLRRLDIYKCEKLMRSPSLSLMGMLMLTDLTIDNVCDGVESFPNKDFALLPPSLTSLTLRNMPSLHTLDCTELLHLTSLQQLEILNCYRLENMAGERLPTTLTQLEIIECPLLEERCRMKHPQIWPKISHIQSILVDHKWI |
| RGA 23 | MTSVLSDCLRVIAEKFLDLTIGEARYLCCFNTIVEEHEREKGKLRAKRKGVMENVNVAKNRAEEIVNDVTVWIQNADNIINDNNEMKRTCFSGWCINCMWQYQRGKELAVKTLEIAELKELNFESVGRAGKLPGVEYHSSQDFIEFESRTSQHKQLLEALKNEENYMVGLHGMGGTGKTTLVQKVGNEVKRSNFFDEVIFTTVSHAPDIRKIQDNIATPLGLKLEEGDQLQRAKKLWSRLTNGERILVILDDVWEELKFEDIGIPSCNNHNACRVLITSRMMSVCNSMSCQSTIDLELLSEEDAKILFEKHTGLRDESPKNLKKLAQQIANECKRSPVAITAIAKSLKHQPPELWKVAYKSLKEFKQIRNVDEDLKIYKCFQVSYENLKDEKAKKLFMLCSLFPEDYEIRVEDLTIFGKGLGIFGDVDSYEAARIEMLTAKRKLLDACLLLKGQEGCVKMHDLVRDAAHWIGNYEIQVIMGSKVHATAKKGTITYLYCQNVKRFCLPNQLDCTKIKILIILCLDKEGFVEMPQAFFEETKDLEVLAISKAENIRGKPSLALPRSIETLKNIRTLCLRGFNLGDISILQKLEILETLELSDCSIIRLPKGMVKLEKLRLLALTCCAIEKNHFEVIGRLSQLEELYVMRSPDRFRWKFDKEVVATIFDRDNIIPTLQRYHIQIGHDSGLYHSVDDSISRALSIEYFDPNSNATIKDLVERAEILHLKRIQGNYTTVTPQLVEAIGGEMNDLINLKLEFCSKIECLIDTNNFSSSIGSIFSKLVKIEIRGMDNLKELCHGPPPSDIFGNLQEMSIYLCHQLHGRLFEGNLNLGRITVFQVEYCRMLTSMFTPSTAASLVLLEELVIEGCKELRNLISYEEEENKQEQIVQHDDNDQKIYGSIFPKLRTFDLRMCDQLEYIITRKDCLGPLSPSDCLSRQSLILRHVREMTLKNCLKIKLLFNLSVARSMLLEELRIKECHSMTSIVTDVGNDESHVTCGSVFPRLKFLSVQDCSQMQYMLGQDHEEHNNDIEIHIYLPELEQLTFSRVPKLISTCSINYNATYPSLKEFCLEECPEFTINSISDFIFHLGARQLADTSTEDIGKMEKHFQTLEKLCIENSDIKGIFSLEELPIIGQQMSSGLQSLKLYNLHELRYTFMGPKHFISLQNIKTLQIEGCSKLKVIFSASVLRSLPQLTYLEINNCEALQRIVEEDDENQSQTNPYSRVVSFPKLVAVVIKCCHSLKSLISVTTFREFPKLELMIIKEASQLDDMFRSEQGDGIPEQKLRLPKLKYLVLMQLPNLVDLSQQMELQNVTYSIIQYCPKLSFDSTTTLENFKNILEDSNIDHEVHRELFEIFDTIMEEAENENPMSETIPQSPIVDEVQDVEVQSAPQRELPCSQIIDEADKEIVAAHDSRMETSSIDLEVITMPYSFPSAILRSKKTQSTKETVEQSLLECPKTENATTTIWLTNSEPSNPSLGPLLIPLQKESSQSPQLVEKQSIDEQITMDQGTIRETNSFNGTPDEHSNLTQNVKISTNGSVSEVVLNKTALAVPSFPELDDSALALISPTSETNVTCSISLPMQRMSSSLLEDIFSKKAKVDHFSNDTENSSSSLILKDELCVYLNMSLEDIIDNNVYNNVERIVNSLAKETTDAFQRNILKDFTNRLKIFKEGVPKAMSTLQSSSEFMSNYENLNMELKAKLNEGQEKIENLETKLSETLAKECTIDMKIKKLINQKNEIVAQKNFLASQLDMYTKVVSKDYENWKGLGEELNSCSDRWLESKEDLAHANASWKILKEILLL |
| RGA 23 RefSeq | MTSVLSDCLRVIAEKFLDLTIGEARYLCCFNTIVEEHEREKGKLRAKRKGVMENVNVAKNRAEEIVNDVTVWIQNADNIINDNNEMKRTCFSGWCINCMWQYQRGKELAVKTLEIAELKELNFESVGRAGKLPGVEYHSSQDFIEFESRTSQHKQLLEALKNEENYMVGLHGMGGTGKTTLVQKVGNEVKRSNFFDEVIFTTVSHAPDIRKIQDNIATPLGLKLEEGDQLQRAKKLWSRLTNGERILVILDDVWEELKFEDIGIPSCNNHNACRVLITSRMMSVCNSMSCQSTIDLELLSEEDAKILFEKHTGLRDESPKNLKKLAQQIANECKRSPVAITAIAKSLKHQPPELWKVAYKSLKEFKQIRNVDEDLKIYKCFQVSYENLKDEKAKKLFMLCSLFPEDYEIRVEDLTIFGKGLGIFGDVDSYEAARIEMLTAKRKLLDACLLLKGQEGCVKMHDLVRDAAHWIGNYEIQVIMGSKVHATAKKGTITYLYCQNVKRFCLPNQLDCTKIKILIILCLDKEGFVEMPQAFFEETKDLEVLAISKAENIRGKPSLALPRSIETLKNIRTLCLRGFNLGDISILQKLEILETLELSDCSIIRLPKGMVKLEKLRLLALTCCAIEKNHFEVIGRLSQLEELYVMRSPDRFRWKFDKEVVATIFDRDNIIPTLQRYHIQIGHDSGLYHSVDDSISRALSIEYFDPNSNATIKDLVERAEILHLKRIQGNYTTVTPQLVEAIGGEMNDLINLKLEFCSKIECLIDTNNFSSSIGSIFSKLVKIEIRGMDNLKELCHGPPPSDIFGNLQEMSIYLCHQLHGRLFEGNLNLGRITVFQVEYCRMLTSMFTPSTAASLVLLEELVIEGCKELRNLISYEEEENKQEQIVQHDDNDQKIYGSIFPKLRTFDLRMCDQLEYIITRKDCLGPLSPSDCLSRQSLILRHVREMTLKNCLKIKLLFNLSVARSMLLEELRIKECHSMTSIVTDVGNDESHVTCGSVFPRLKFLSVQDCSQMQYMLGQDHEEHNNDIEIHIYLPELEQLTFSRVPKLISTCSINYNATYPSLKEFCLEECPEFTINSISDFIFHLGARQLADTSTEDIGKMEKHFQTLEKLCIENSDIKGIFSLEELPIIGQQMSSGLQSLKLYNLHELRYTFMGPKHFISLQNIKTLQIEGCSKLKVIFSASVLRSLPQLTYLEINNCEALQRIVEEDDENQSQTNPYSRVVSFPKLVAVVIKCCHSLKSLISVTTFREFPKLELMIIKEASQLDDMFRSEQGDGIPEQKLRLPKLKYLVLMQLPNLVDLSQQMELQNVTYSIIQYCPKLSFDSTTTLENFKNILEDSNIDHEVHRELFEIFDTIMEEAENENPMSETIPQSPIVDEVQDVEVQSAPQRELPCSQIIDEADKEIVAAHDSRMETSSIDLEVITMPYSFPSAILRSKKTQSTKETVEQSLLECPKTENATTTIWLTNSEPSNPSLGPLLIPLQKESSQSPQLVEKQSIDEQITMDQGTIRETNSFNGTPDEHSNLTQNVKISTNGSVSEVVLNKTALAVPSFPELDDSALALISPTSETNVTCSISLPMQRMSSSLLEDIFSKKAKVDHFSNDTENSSSSLILKDELCVYLNMSLEDIIDNNVYNNVERIVNSLAKETTDAFQRNILKDFTNRLKIFKEGVPKAMSTLQSSSEFMSNYENLNMELKAKLNEGQEKIENLETKLSETLAKECTIDMKIKKLINQKNEIVAQKNFLASQLDMYTKVVSKDYENWKGLGEELNSCSDRWLESKEDLAHANASWKILKEILLL |

**Supplementary Figure 1. The electrophoresis gel picture for checking genomic contamination in cDNA samples, the well contents are shown in the table below.** (PBA Seamer, PBA HatTrick, Kyabra, ICC 3996; T= treated with pathogen, U= untreated. And each sample has three replicates. So ST2-1 = PBA Seamer_treated with pathogen_2hpi_rep1, same as others**)**

**
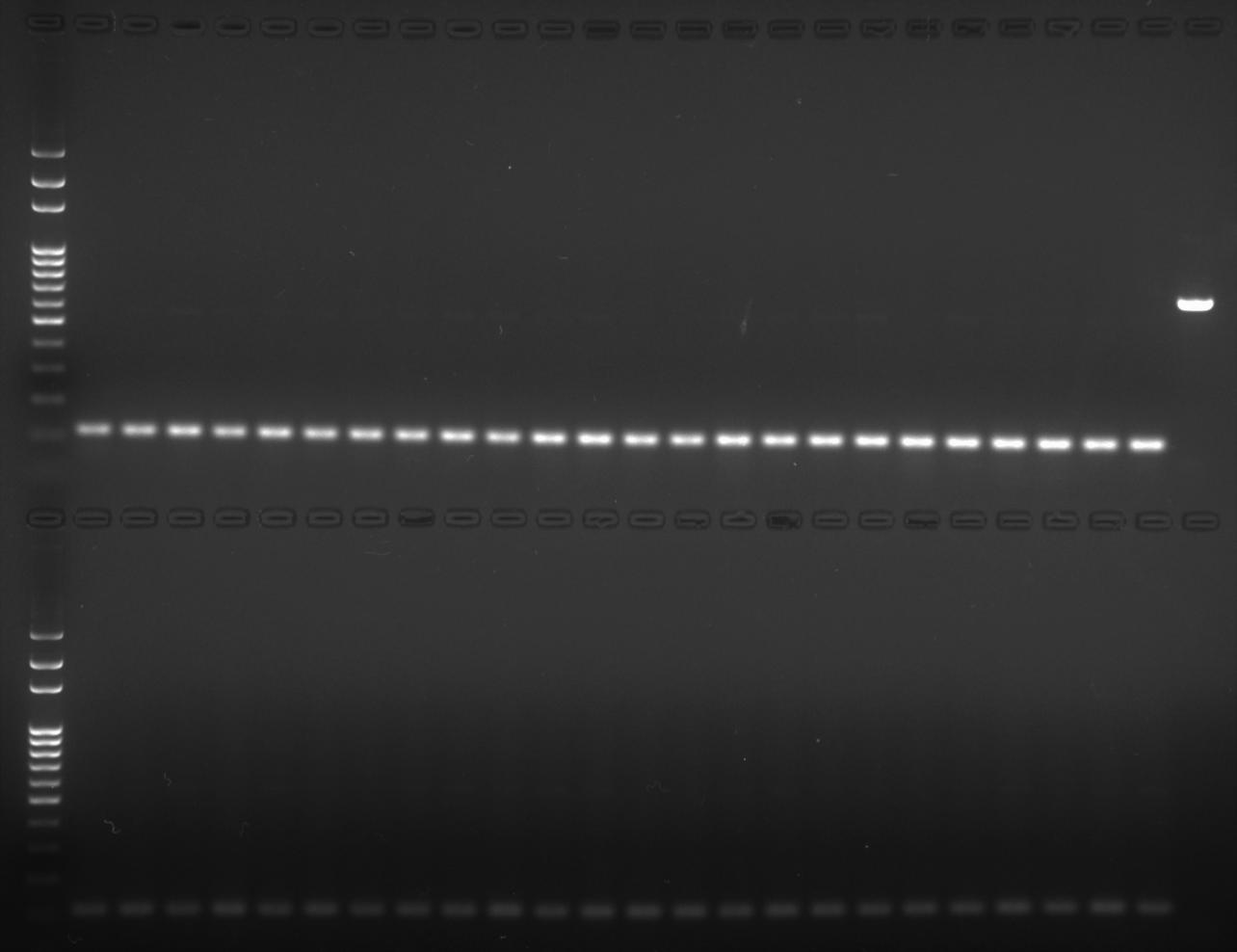
**

| **Content** | **Well** | **Row** |
| --- | --- | --- |
| 100bp Ladder | 1 | 1 |
| ST2-1 | 2 | 1 |
| ST6-1 | 3 | 1 |
| ST2-2 | 4 | 1 |
| ST6-2 | 5 | 1 |
| ST2-3 | 6 | 1 |
| ST6-3 | 7 | 1 |
| HT2-1 | 8 | 1 |
| HT6-1 | 9 | 1 |
| HT2-2 | 10 | 1 |
| HT6-2 | 11 | 1 |
| HT2-3 | 12 | 1 |
| HT6-3 | 13 | 1 |
| KT2-1 | 14 | 1 |
| KT6-1 | 15 | 1 |
| KT2-2 | 16 | 1 |
| KT6-2 | 17 | 1 |
| KT2-3 | 18 | 1 |
| KT6-3 | 19 | 1 |
| IT2-1 | 20 | 1 |
| IT6-1 | 21 | 1 |
| IT2-2 | 22 | 1 |
| IT6-2 | 23 | 1 |
| IT2-3 | 24 | 1 |
| IT6-3 | 25 | 1 |
| gDNA | 26 | 1 |
| 100bp Ladder | 1 | 2 |
| ST24-1 | 2 | 2 |
| SU2 | 3 | 2 |
| ST24-2 | 4 | 2 |
| SU6 | 5 | 2 |
| ST24-3 | 6 | 2 |
| U24 | 7 | 2 |
| HT24-1 | 8 | 2 |
| HU2 | 9 | 2 |
| HT24-2 | 10 | 2 |
| HU6 | 11 | 2 |
| HT24-3 | 12 | 2 |
| HU24 | 13 | 2 |
| KT24-1 | 14 | 2 |
| KU2 | 15 | 2 |
| KT24-2 | 16 | 2 |
| KU6 | 17 | 2 |
| KT24-3 | 18 | 2 |
| KU24 | 19 | 2 |
| IT24-1 | 20 | 2 |
| IU2 | 21 | 2 |
| IT24-2 | 22 | 2 |
| IU6 | 23 | 2 |
| IT24-3 | 24 | 2 |
| IU24 | 25 | 2 |
| NTC | 26 | 2 |
